# A Machine Learning Approach for Continuous Mining of Nonidentifiable Smartphone Data to Create a Novel Digital Biomarker Detecting Generalized Anxiety Disorder: Prospective Cohort Study

**DOI:** 10.2196/38943

**Published:** 2022-08-30

**Authors:** Soumya Choudhary, Nikita Thomas, Sultan Alshamrani, Girish Srinivasan, Janine Ellenberger, Usman Nawaz, Roy Cohen

**Affiliations:** 1 Department of Research Behavidence, Inc. New York, NY United States; 2 Department of Data Science Behavidence, Inc. New York, NY United States

**Keywords:** digital phenotyping, machine learning, mental health, profiling metric, smartphone data, anxiety assessment, mining technique, algorithm prediction, digital marker, behavioral marker, anxiety

## Abstract

**Background:**

Anxiety is one of the leading causes of mental health disability around the world. Currently, a majority of the population who experience anxiety go undiagnosed or untreated. New and innovative ways of diagnosing and monitoring anxiety have emerged using smartphone sensor–based monitoring as a metric for the management of anxiety. This is a novel study as it adds to the field of research through the use of nonidentifiable smartphone usage to help detect and monitor anxiety remotely and in a continuous and passive manner.

**Objective:**

This study aims to evaluate the accuracy of a novel mental behavioral profiling metric derived from smartphone usage for the identification and tracking of generalized anxiety disorder (GAD).

**Methods:**

Smartphone data and self-reported 7-item GAD anxiety assessments were collected from 229 participants using an Android operating system smartphone in an observational study over an average of 14 days (SD 29.8). A total of 34 features were mined to be constructed as a potential digital phenotyping marker from continuous smartphone usage data. We further analyzed the correlation of these digital behavioral markers against each item of the 7-item Generalized Anxiety Disorder Scale (GAD-7) and its influence on the predictions of machine learning algorithms.

**Results:**

A total of 229 participants were recruited in this study who had completed the GAD-7 assessment and had at least one set of passive digital data collected within a 24-hour period. The mean GAD-7 score was 11.8 (SD 5.7). Regression modeling was tested against classification modeling and the highest prediction accuracy was achieved from a binary XGBoost classification model (precision of 73%-81%; recall of 68%-87%; *F*_1_-score of 71%-79%; accuracy of 76%; area under the curve of 80%). Nonparametric permutation testing with Pearson correlation results indicated that the proposed metric (Mental Health Similarity Score [MHSS]) had a colinear relationship between GAD-7 Items 1, 3 and 7.

**Conclusions:**

The proposed MHSS metric demonstrates the feasibility of using passively collected nonintrusive smartphone data and machine learning–based data mining techniques to track an individuals’ daily anxiety levels with a 76% accuracy that directly relates to the GAD-7 scale.

## Introduction

### Background and Rationale

Anxiety is one of the leading causes of mental health disability around the world [1]. It includes feelings of excessive worry and negative thoughts, accompanied by physical symptoms such as heart palpitations and increased blood pressure [2]. Anxiety is also associated with a high degree of functional impairment [3] leading to poor quality of life [4] and high health care utilization [5]. Despite being one of the leading causes of mental health disability (1 in 4 people according to the World Mental Health Survey [6]), the detection of generalized anxiety disorder (GAD) is very low in primary care settings [7-9]. These challenges stem from the problems regarding diagnostic processes and inaccuracies [8,10-16] as well as overlapping comorbidities [9,17,18] and physical symptomatology [5,19]. The diagnosis is also vulnerable to the observer’s state of mind [20] and biased self-perception [21] of symptoms. Whether it is the diagnosis of GAD as a singular condition or as a comorbidity, the validity of the diagnostic classifications and instruments in themselves has been rigorously debated. Newson et al [22] highlighted the heterogeneity in DSM-5 classification, where it failed to diagnose a specific disorder from random. Zimmerman et al [23] demonstrated how a physician can diagnose depression and its comorbidities in 227 different ways and Phillips [15] has highlighted the ambiguities in DSM-5 criteria for disorder classification. A recent analysis [10] of eHealth data, patient records, and physician reports in psychiatric cases has highlighted the presence of diagnostic errors in two-thirds of the sample.

With the advancement of technology, researchers have employed multisource data and advanced data analysis techniques to refine and improve mental health diagnosis. One such opportunity to use an upcoming method to improve screening of anxiety is to harness the power of smartphones using the principles of digital phenotyping [24]. Digital phenotyping is a novel computational approach that relies on real-time quantification of human behavior through continuous monitoring of digital biomarkers [25-27]. Mobile and wearable digital devices offer the opportunity to track a multitude of parameters such as mobility (through GPS and accelerometer) [28,29], societal interactions [30] (number of calls, voice tone detection, number of messages sent), digital interactions (access to certain apps), phone usage frequency (screen turned on/off) [27], and health monitoring parameters (heart rate, blood pressure, and oxygen saturation) [31]. However, most digital phenotyping approaches present limited applicability due to the lack of standardized data processing approach for big data exploitation and lack of a specific pattern of unique features for complex mental conditions such as anxiety disorder.

### Previous Findings

Smartphones hold huge potential in redefining the ability to understand mental health behavior. Sensors embedded in smartphones allow for both passive and continuous data collection, which enhances the possibility of understanding human behavior daily [32-34]. Longitudinal monitoring of passive sensors and phone usage has been linked to tracking mental health behavioral trends [24]. Digital phenotyping of mental health has proven successful in dealing with the challenges associated with a diagnosis such as biases in self-reporting and lack of time in primary care settings, thus paving the way for new and novel methods of screening and monitoring [35].

Most previous studies have focused on using digital phenotyping and passive sensor data to predict social anxiety rather than generalized anxiety [28,29,32,36]. In addition, the passive data used in previous research were intrusive of the users’ privacy and collected identifiable data points such as GPS, audio, message logs, and Bluetooth. Jacobson et al [29] demonstrated that sensor data such as accelerometer, call log, and text message data from smartphones could predict social anxiety symptom severity. Another study found that people with high social anxiety had much lower call and text message logs, and used more health and fitness apps and less camera apps as compared with the low social anxiety group [36]. A clinical review on digital phenotyping and the mental health of college students found that sensors such as accelerometer, Bluetooth, and social information can help in understanding clinical symptomatology [37]. By contrast, Meyerhoff et al [28] found that GPS-based sensor features can be useful in predicting depression severity, but it was not significant in predicting anxiety. Other studies that have researched generalized anxiety have been grouped along with other disorders such as depression and social anxiety. The sensors that have been utilized included location sampled every 5 minutes, call and message log data, duration, and length. Interestingly, these studies also found that there was no significant relationship between GAD and location sensors [28,38]. A more recent study investigated how features extracted from smartphones can be used to predict GAD, social anxiety disorder, and depression. The authors found that their machine learning models and features were able to predict social anxiety disorder and depression severity but not GAD [25]. Such findings have paved the way to explore more ways to map generalized anxiety using nonintrusive and nonidentifiable smartphone data.

### Study Objective

In this study a novel mental behavioral profiling metric, derived from smartphone usage, is defined for the identification and tracking of GAD. The accuracy of this metric is evaluated in relation to the standardized anxiety assessment protocol using the 7-item Generalized Anxiety Disorder Scale (GAD-7) questionnaire scoring.

## Methods

### Data Collection Procedure

Participants were recruited via an advertisement through social media campaigns on Facebook and Google. Research has shown that this is an effective means of recruitment and provides more generalizability than a clinic-recruited study [39]. Interested participants responded to the advertisement by reading about the study and signing the informed consent form. They then downloaded the “Behavidence Research App” from the Google Play store and filled in a demographic questionnaire, followed by the GAD-7 scale. These data were collected at a single time point only during the onboarding process. The app continued to passively collect nonintrusive data from the smartphone such as screen time and app usage, with no engagement requirement from the user. There was absolutely no private information collected, making this solution completely nonintrusive and secure. Data were collected between October 2021 and January 2022. The participants were informed about the type of nonidentifiable passive data collected in the consent form.

### Inclusion/Exclusion Criteria

A total of 238 globally distributed users responded to the online advertisement. The inclusion criteria were (1) participants should be over 18 years of age; (2) participants must be able read, speak, and write in English; and (3) participants must have an Android smartphone. Of the enrolled participants, 229 completed the entire on-boarding process. There were no restrictions on gender, ethnicity, or the participant’s location.

### Measure

#### Generalized Anxiety Disorder Screening

The GAD-7 scale [40] is a self-report scale with 7 items for screening nonspecific anxiety in primary care settings. It also indicates the severity of GAD. The items of the scale are rated on a Likert scale ranging from “0=Not at all” to “3=Nearly every day.” The scores range from 0 to 21. This questionnaire has good psychometric properties within community and psychiatric samples [41] and has also been established in previous research [42].

#### Digital Data Collection Through Behavidence

**Figure 1:**
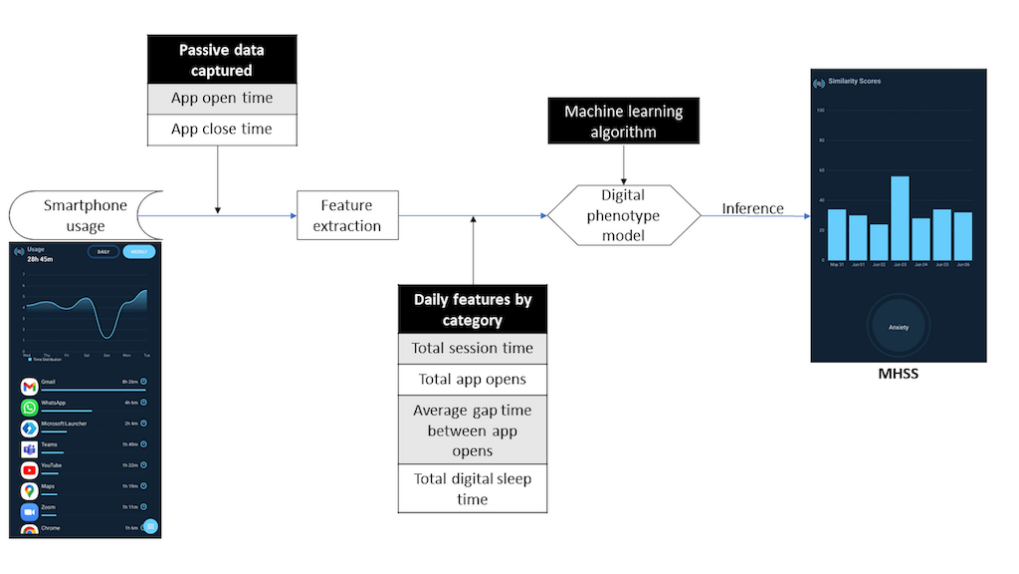
The Behavidence solution workflow demonstrates key steps in the creation of a mental health similarity score for anxiety. MHSS: Mental Health Similarity Score.

Behavidence [43] is a mental health screening app that passively collects personal smartphone device usage. The app works as a digital profiling solution and can be downloaded from the Google Play Store. There is zero response burden and no collection of any identifiable information. The app was developed for smartphones running Android version 5 or higher. It requires internet connectivity to receive outcomes of data analysis but does not require an active internet connection to collect the data. As the app runs in the background, the participant must provide “Battery Optimization” and “Usage Data Access” permission, obtained during the log-in process. The main screen of the app displays a Mental Health Similarity Score (MHSS), which is inferred from the user’s digital behavior. The MHSS displays how similar the user’s digital behavior is to someone else’s digital behavior who has a diagnosis of anxiety. The similarity score is generated once every 24 hours and has a range of 0%-100%. The app also shows the user their weekly history of daily similarity scores. The workflow of the solution is shown in Figure 1. Data access is managed by multifactor federated authentication and controlled through role-based privileges. Policies are created to manage access for each user, user group, or role. The data pipeline is encrypted end-to-end and orchestrated under enterprise-grade privacy and compliance certification. Data are protected while in-transit via secure socket layer/transport layer security (SSL/TLS) and client-side encryption. Server-side encryption with managed keys is used before storing the data. The application is Health Insurance Portability and Accountability Act (HIPAA) and General Data Protection Rule (GDPR) compliant.

### Data Mining

#### App Categorization

The total number of apps used by the participants in this study exceeded 50,000 unique apps. To be able to understand and measure features related to each app, we categorized them into 11 categories as follows: Category 0 for nonofficial or unregulated apps, Category 1 for social interaction apps, Category 2 for passive information consumption apps, Category 3 for active messaging and communications apps, Category 4 for educational apps, Category 5 for navigation utilities, Category 6 for general utilities, Category 7 for recreational and photo processing apps, Category 8 for commerce apps, Category 9 for health and fitness–related apps, Category 10 for games, and lastly, Category 11 for miscellaneous.

#### Feature Extraction Using Passive Smartphone Data

Passive collection of raw nonidentifiable smartphone data starts after the user completes the GAD-7 questionnaire. Seven days of retrograde data are automatically available after a new user log-in, and data are continuously streamed to the back end until the user logs out or deletes the app. The raw data collected include the time in milliseconds of Coordinated Universal Time (UTC) in which a user opens a particular app and the time a user closes that app. From these raw data, behavioral insights used as features for the machine learning algorithms are drawn on a 24-hour basis. For example, the total session time on a phone is calculated by summing the total number of milliseconds the user spends on each app he/she opens, between 12 AM in the user’s local time zone to 11:59 PM that day. Incomplete 24-hour data are omitted from the feature engineering process and may be attributed to network disconnection of the user’s Android device. No users in this study had gaps of incomplete 24-hour data within consecutive days of collection. Mobile apps were also binned into specific app categories (see the “App Categorization” section) for further insights into digital behavior. Frequency and duration of each app category are calculated daily to indicate where the user spends the most time on their mobile device (ie, shopping, gaming, online dating, communication). Therefore, a total of 34 features were extracted from the original raw data (full list of features are listed in Multimedia Appendix 1).

#### Data Preparation and Model Setup

A single independent observation in this study constituted 24 hours (user’s time zone) of raw data transmitted by the Behavidence App to the back end secure cloud system. Therefore, an individual with anxiety that had 15 days of full passively acquired data was considered to have 15 separate anxiety-labeled observations. To evaluate the models, we reported on different accuracy metrics using 5-fold cross-validation. With 5-fold cross-validation, the data set was split into 5 groups where models were trained on 4 groups and validated on the left-out group. The process was repeated 5 times so that each sample was used for training and validation only once. The Amazon Web Services platform (Amazon.com, Inc.) was used as data storage while the data processing, feature engineering, model training, and poststatistical analysis were written in Python 3.8 programming language (Python Software Foundation). Packages used include scipy, stats models, net neurotools, and scikit-learn.

### Modeling and Postanalysis

#### Machine Learning to Predict Generalized Anxiety

To explore the efficacy of digital behavioral markers in detecting generalized anxiety, regression and classification models were implemented. First, a random forest algorithm was used to create a nonlinear multiple regression fit for the passive digital data corresponding to the total possible score of 21 for the GAD-7 scale. The purpose of this model was to infer what GAD-7 score a user would obtain based on his/her phone usage. For the classification models, 4 different machine learning algorithms were compared to produce the highest overall prediction accuracy. The algorithms compared include random forest, K-nearest neighbors, logistic regression, and XGBoost. The multiclass GAD-7 model is intended to classify participants who scored 15+ (severe ), 10-14 (moderate), 5-9 (mild), and <5 (no diagnosis) to detect the progression into severe anxiety. The binary GAD-7 model is intended to classify participants who scored 15+ (severe) on the GAD-7 against those who scored <5 (ie, having no indication of anxiety).

#### Correlation-Based Analysis

Further analysis on specific items from the GAD-7 was conducted to determine which symptoms of anxiety can be understood from the passively collected digital data. Each of the 7 questions was tested against the MHSS obtained from the top-performing GAD-7 model and calculated on the day each user answered the questionnaire. This testing was performed to determine the existence of a relationship between the digital behaviors collected from the Behavidence app and each question of GAD-7. Nonparametric permutation tests were performed to determine the significance of the Pearson correlation, with the number of permutations set to 1000. Permutation testing was used to better estimate the population’s distribution, by not assuming a normal distribution (nonparametric), and to ultimately determine extremities more accurately, by leveraging resampling, so that *P* values indicate the true probability that the Pearson correlation coefficient calculated is not by chance. As the MHSS is derived from the 34 passive digital features, further correlation between specific items from the GAD-7 questionnaire and each of the features was assessed to determine whether the digital biomarker in this study could be mapped to the symptoms of GAD that the specific items are targeting.

### Ethics Approval

The advertisement, informed consent, and the study protocol were approved by the independent Western Institutional Board Copernicus Group (WIRB-CG) institutional review board (Approval Number 20216225).

## Results

### Participants

Self-reported demographic data from the 229 participants (Table 1) show that 85 (37.1%) identified as females, 142 (62%) identified as males, and 2 (0.9%) identified as nonbinary or preferred not to disclose their gender. For the participants’ age distribution, 102 (44.5%) were aged between 18 and 25, 66 (28.8%) between 26 and 35, 56 (24.5%) between 36 and 55, and 5 (2.2%) between 56 and 64. A majority of the participants that completed the questionnaire were of Asian race (104/229, 45.4%), and had education levels between some college diploma and a bachelor’s degree (158/229, 69%). The participants in this study were from different locations around the globe. Most were in Asia (84/229, 36.7%) followed by Africa (76/229, 33.2%). The remaining participants were from America, Europe, and Australia.

**Table 1:** Demographic data of the participants who answered the GAD-7^a^ questionnaire (n=229).

Category		Values, n (%)
**Age, years**		
	18-25	102 (44.5)
	26-35	66 (28.8)
	36-55	56 (24.5)
	56-64	5 (2.2)
**Gender**		
	Male	142 (62.0)
	Female	85 (37.1)
	Prefer not to say	2 (0.9)
**Race**		
	Asian	104 (45.4)
	Black (African/Caribbean)	40 (17.5)
	White	61 (26.6)
	Mixed	11 (4.8)
	Other/prefer not to say	13 (5.7)
**Education**		
	Lower secondary/middle school (grades 7-9)	2 (0.9)
	Higher secondary (grades 10-12)	35 (15.3)
	Some college/university/diploma	74 (32.3)
	Bachelor’s degree	84 (36.7)
	Master’s degree	28 (12.2)
	Professional/PhD	6 (2.6)
**Time zone**		
	Africa	76 (33.2)
	Americas	9 (3.9)
	Asia	84 (36.7)
	Australia	1 (0.4)
	Europe	13 (5.7)
	Other^b^	46 (20.1)

^a^GAD-7: 7-item Generalized Anxiety Disorder Scale.

^b^All the other time zones that were unspecified.

### GAD-7 Distribution Among Participants

Table 2 represents the distribution of the 229 recruits and their GAD-7 scoring. The GAD-7 was completed at the start of recruitment at a single time point during this study, which spanned from October 2021 to January 2022. The distribution of the GAD-7 scores was as follows: 23/229 (10%) were none with GAD-7 scoring less than 5, while 206/229 (89.9%) showed signs of anxiety by scoring between “mild” and “severe.” The mean GAD-7 score was 11.8 (SD 5.7).

**Table 2:** Distribution of participants’ contribution to the GAD-7^a^ responses (n=229).

GAD-7 category and scores		Participants, n (%)
**None**		23 (10)
	0	10
	1	2
	2	3
	3	3
	4	5
**Mild**		61 (26.6)
	5	13
	6	6
	7	19
	8	11
	9	12
**Moderate**		64 (27.9)
	10	10
	11	10
	12	13
	13	17
	14	14
**Severe**		81 (35.4)
	15	14
	16	10
	17	11
	18	14
	19	7
	20	10
	21	15

^a^GAD-7: 7-item Generalized Anxiety Disorder Scale.

As seen in Table 3 16% (14/88) of self-reported healthy “none” group participants scored “none” on the GAD-7, whereas the greatest percentage (29/88, 33%) of participants in this group scored “moderate” anxiety. Table 3 also shows that 52% (13/25) of participants with self-reported anxiety had severe anxiety on the GAD-7. Further, 61% (31/51) of participants with self-reported depression had “severe” anxiety and only 2% (1/51) had no signs of anxiety.

**Table 3:** Distribution of GAD-7^a^ scoring categories for self-reported participants.

Self-reported diagnosis	None, n (%)	Mild, n (%)	Moderate, n (%)	Severe, n (%)
a. None (n=88)	14 (16)	15/88 (17)	29/88 (33)	22/88 (25)
b. Anxiety (n=25)	N/A^b^	5/25 (20)	7/25 (28)	13/25 (52)
c. Depression (n=51)	1/51 (2)	6/51 (12)	13/51 (25)	31/51 (61)

^a^GAD-7: 7-item Generalized Anxiety Disorder Scale.

^b^N/A: no participants with a self-reported diagnosis of anxiety scored “none” on the GAD-7 questionnaire.

### Evaluation of Models

#### Overview

The aim of the study was to evaluate the accuracy of the MHSS metric to identify GAD. The binary classification XGBoost model achieved a prediction accuracy of 76% compared with 50% by the multiclass classification XGBoost model and regression (root-mean-squared error [RMSE] 4.508). The recall scores for the binary model were 68% for the “none” group and 87% for the “anxiety group.” Using the multiclass XGBoost model the best recall scores achieved were 41%, 63%, 38%, and 52% for the “none,” “mild,” “moderate,” and “severe” groups, respectively. The reported results are from the 5-fold cross-validation of data.

#### Regression Model Assessment

**Figure 2:**
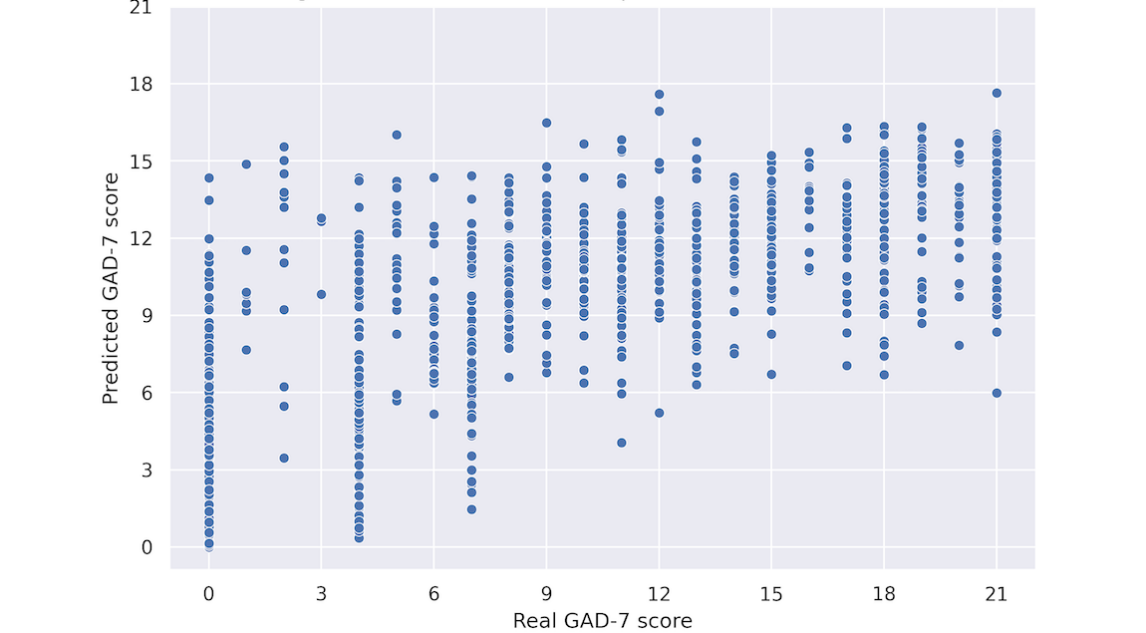
Random forest regression: real GAD-7 score versus predicted GAD-7 score (correlation: 0.65597). GAD-7: 7-item Generalized Anxiety Disorder Scale.

Figure 2 shows the random forest regression model–predicted GAD-7 score plotted against the actual GAD score. The range of predicted values in the lower scores (0-7) is quite high, distributing around 75% of all possible scores. The RMSE for this model is 4.508 with an *R*^2^ value of 0.4282.

#### Multiclass Classification

The multiclass classification model, trained on all severity group classes, none (GAD-7*<*5), mild (5≤GAD-7*<*10), moderate (10≤GAD-7*<*15), and severe (GAD-7≥20), that achieved the highest prediction accuracy was using XGBoost followed by the random forest algorithm. Result metrics from the 4 algorithm comparisons are presented in Table 4. The GAD-7 multiclass XGBoost model achieved a precision of 40%-62%, recall of 38%-63%, *F*_1_-score of 39%-61%, and overall accuracy of 50%. Sensitivity for severe anxiety was 52% and specificity was 74%.

**Figure 3:**
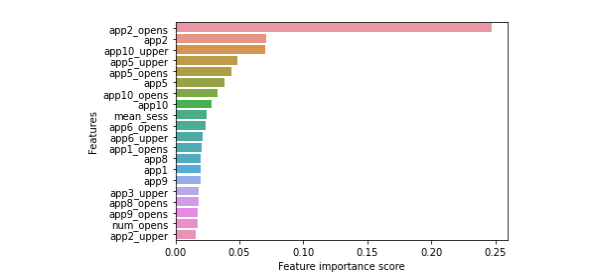
Feature importance of the GAD-7 multiclass XGBoost model. GAD-7: 7-item Generalized Anxiety Disorder Scale.

The Gini impurity plot of each feature shows the top features that the multiclass XGBoost model considers when differentiating between all the possible groups (Figure 3). The 3 most important features in this classifier were the number of times “passive information consumption” apps were opened within the 24-hour period (app2_opens), mean session time within a 24-hour period in “passive information consumption” apps (app2), and the number of times “games” apps were opened with session lengths greater than 1 SD from the mean (app10_upper).

Analysis of variance was performed to determine the difference among means of the 4 different cohorts (ie, none, mild, moderate, and severe) for the top 3 Gini important features. For the feature summing the total number of times “passive information consumption” apps were opened, *F*_4,2619_=63.40 and *P*=.44. For the average session time on passive information consumption apps, *F*_4,2619_=5.23 and *P*=.002. Finally, for the number of times “games” apps were opened with session lengths greater than 1 SD from the mean, *F*_4,2619_=60.22 and *P*=.26. In addition, Tukey post hoc test for pairwise comparison was performed with Cohen *d* effect size. Detailed results can be found in Multimedia Appendix 2.

**Table 4:** Multiclass classification accuracy metrics of all algorithms tested in this study (random forest, k-nearest neighbors, logistic regression, XGBoost) using 5-fold cross-validation.

Class			GAD-7^a^ multiclass RF model, %		GAD-7 multiclass K-nearest neighbors model, %		GAD-7 multiclass logistic regression model, %		GAD-7 multiclass XGB model, %
Accuracy			48		29		38		50
Area under the curve			69		53		56		71
**Precision**									
	None	64		27		19		62	
	Mild	58		33		41		60	
	Moderate	37		27		39		41	
	Severe	39		27		33		40	
**Recall**									
	None	41		22		77		41	
	Mild	58		34		24		63	
	Moderate	41		28		0.4		38	
	Severe	48		29		29		52	
***F*_1_-score**									
	None	50		24		53		50	
	Mild	58		34		29		61	
	Moderate	39		28		0.6		39	
	Severe	43		28		31		45	

^a^GAD-7: 7-item Generalized Anxiety Disorder Scale.

#### Binary Classification

The random forest classification model, which trained on 2 classes (none vs severe anxiety) and 34 features with the number of trees set to 50, achieved a precision of 79%-70%, recall of 59%-86%, *F*_1_-score of 68%-78%, an overall accuracy of 74%, and area under the curve (AUC) of 78% (Table 5). The binary logistic regression model achieved a precision of 55%-56%, recall of 28%-80%, *F*_1_-score of 37%-66%, an overall accuracy of 55%, and AUC of 57%. The binary K-nearest neighbors model, with k set to 17 according to optimized parametric tuning, achieved a precision of 59%-60%, recall of 46%-73%, *F*_1_-score of 52%-66%, an overall accuracy of 60%, and AUC of 62%. Finally, the binary XGBoost model was the one with the highest accuracy, which achieved a precision of 81%-73%, recall of 68%-87%, *F*_1_-score of 71%-79%, an overall accuracy of 74%, and AUC of 78%. This model can successfully differentiate between “none” and “severe” anxiety.

**Figure 4:**
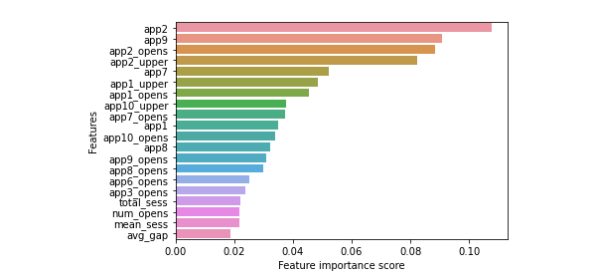
Feature importance of the GAD-7 Binary XGBoost model. GAD-7: 7-item Generalized Anxiety Disorder scale.

In this experiment, the best performing classification algorithm is the XGBoost, which consists of 50 trees that use the Gini criterion to measure the quality of a split with no maximum depth and a minimum of 2 samples per split. The model was further analyzed by plotting Gini impurity values of each feature because this method was used as the splitting criterion of the classification trees when determining the none and severe anxiety groups. As seen in Figure 4, the top 3 passive digital features were mean session time within a 24-hour period in the “passive information consumption” apps (app category 2), mean session time within a 24-hour period in the “health and fitness” apps, and the number of times “passive information consumption” apps were opened within the 24-hour period (app2_opens). The *t* test (unpaired) results indicated statistical significance on all 3 of the top features (Table 6). The effect size ranges from low to high, with the total number of times social interaction apps opened having the greatest effect size (Table 6).

**Table 5:** Accuracy metrics of all binary classification models trained in this study (random forest, k-nearest neighbors, logistic regression, and XGBoost) using 5-fold cross-validation.

Class		GAD-7^a^ binary RF model, %	GAD-7 binary K-nearest neighbors model, %	GAD-7 binary logistic regression model, %	GAD-7 binary XGB model, %
Accuracy		74	60	55	76
AUC^b^		78	62	57	80
**Precision**					
	None	79	59	55	81
	Anxiety	70	60	56	73
**Recall**					
	None	59	46	28	68
	Anxiety	86	73	80	87
***F*_1_-score**					
	None	68	52	37	71
	Anxiety	78	66	66	79

^a^GAD-7: 7-item Generalized Anxiety Disorder Scale.

^b^AUC: area under the curve.

**Table 6:** Nonparametric *t* tests on the top 3 Gini importance features of the GAD-7^a^ binary XGBoost model.

Feature description	None, mean (SD)	Severe, mean (SD)	*P* value	Cohen *d*
App category 2, average session time on passive information consumption apps (minutes)	0.22 (1.03)	0.08 (0.44)	.002	0.18
App category 9, average session time on Health and Fitness apps (minutes)	0.39 (0.93)	0.60 (1.49)	.003	–0.16
App category 2 opens, total number of times passive information consumption apps were opened (count)	0.51 (1.05)	0.43 (2.46)	.45	0.041

^a^GAD-7: 7-item Generalized Anxiety Disorder Scale.

### Correlations of GAD-7 Items

Each GAD-7 item was tested using nonparametric permutation testing with Pearson correlation against MHSS on the day that the GAD-7 was filled (Table 7). The highest correlated items belonged to Items 1, 3, and 7: (1) “Feeling nervous, anxious, or on edge” had a correlation of 0.54 (*P*<.001), (3) “Worrying too much about different things” had a correlation of 0.59 (*P*<.001), and (7) “Feeling afraid, as if something awful might happen” had a correlation of 0.55 (*P*<.001).

**Table 7:** Nonparametric permutation testing with Pearson correlation of GAD-7^a^ items against MHSS^b^ on the day the questionnaire was filled.

Item	Pearson correlation, *r*	*P* value
1: “Feeling nervous, anxious, or on edge”	0.54	*<*.001
2: “Not being able to stop or control worrying”	0.5	*<*.001
3: “Worrying too much about different things”	0.59	*<*.001
4: “Trouble relaxing”	0.48	*<*.001
5: “Being so restless that it’s hard to sit still”	0.32	*<*.001
6: “Becoming easily annoyed or irritable”	0.5	*<*.001
7: “Feeling afraid, as if something awful might happen”	0.55	*<*.001

^a^GAD-7: 7-item Generalized Anxiety Disorder Scale.

^b^MHSS: Mental Health Similarity Score.

## Discussion

### Principal Findings

Smartphone technology has certainly become a primary platform not only for communication but also to receive, manage, and share multiple kinds of data. Recently, the application of smartphones and their sensing capabilities have demonstrated huge potential in health information acquisition and analysis [25-30,34-38]. Mining smartphone data to represent digital behavior can be used for delivering informed clinical decisions and early risk stratification of mental health disorders. Through this study, we demonstrate the application of digital phenotyping in the identification and remote monitoring of GAD.

A novel mental behavioral profiling metric called MHSS was derived by engineering 34 digital features to serve as a marker for GAD. This was accomplished using smartphone usage data mined in a passive manner without the use of any private information. The smartphone usage data comprised active app usage time and frequency collected through the Behavidence app for an average period of 14 days per user. A single observation that consists of 24 hours of smartphone usage data had a typical size of 30 KB. During the course of the study, the engagement with the Behavidence app (number of times the app was opened per day) had an average of 0.78%, highlighting the benefit of zero respondent burden. Answering the GAD-7 questionnaire was only for the purpose of training the models and testing its performance. Models created in the study explored the ability of the MHSS to predict the GAD-7 outcome at 3 levels of granularity. The regression model explored the conformance of MHSS to GAD-7 on an individual score level (0-21) and achieved an RMSE of 4.508. The multiclass classification model encoded 4 levels of anxiety severity with an overall accuracy of 50%, whereas the binary classification model distinguished individuals with severe anxiety from the ones without any anxiety with an overall accuracy of 76%.

Although there can be a substantial within-subject variability in scoring across time as mentioned by Meyerhoff et al [28], the reported SD for GAD-7 (3.50) is less than the RMSE achieved in this study. In a clinical use case, the GAD-7 score–based anxiety category is more relevant than the individual scores. Interrater reliability of anxiety disorder diagnosis is shown to have a κ value of 0.20 [44]. A key performance indicator for MHSS would be its ability to differentiate individuals across the anxiety categories with an accuracy over 70%. Each anxiety category (ie, none, mild, moderate, and severe) has a range of 4 points in the GAD-7 scale. As the RMSE in this regression model exceeds this range, this model would result in very low accuracy of anxiety category prediction.

The GAD-7 multiclass model achieved an overall accuracy of 50%, with a sensitivity of 63%, 37%, 41%, and 52% and specificity of 80%, 84%, 93%, and 74% for the none, mild, moderate, and severe classes, respectively. Prior studies performed in primary care clinics have noted that a cut-off score of 10 or higher on the GAD-7 scale has a sensitivity of 89% and specificity of 82% [45]. Although GAD-7 may be particularly useful in assessing symptom severity, a score of 10 or greater on the GAD-7 is most reliable for identifying cases of GAD. This supports the case for developing a binary classification model as an effective screening tool. With the available number of participants in the study, the statistical power for differentiating participants with severe anxiety from ones without anxiety using the digital phenotype as a marker was the strongest (76%). Based on testing various modeling algorithms including random forest, logistic regression, K-nearest neighbors, and RF, the GAD-7 binary XGBoost model achieved 76% accuracy with a sensitivity of 62% and specificity of 86%. These accuracy levels are higher than published results that use intrusive markers to predict generalized and social anxiety disorder [25], or that have used physiological markers to predict anxiety severity [46]. Along with the accuracy levels, sensitivity and specificity results for the GAD-7 binary model are also higher than studies done by Nemesure et al [47] and Fukazawa et al [48], which used binary classification for prediction of anxiety.

One of the key findings was the higher use of certain app categories such as “passive information consumption apps,” “games,” and “health and fitness” among participants with anxiety as compared with those without. Feature importance analysis has been performed by various previous studies, and they have demonstrated the usefulness of knowing these predictors [49]. Previous studies have stated various features such as daily screen time [25] as useful predictors. This study highlights certain app categories as important predicting features, allowing a deep dive into the digital usage patterns of people with and without anxiety. Whether the increased usage of such apps is a result or a cause of elevated anxiety is a topic for further exploration.

The correlation analysis performed between the items of the GAD-7 scale found that the highest correlated items were 1, 3, and 7. This has been a very interesting finding because the 2-factor structure of the GAD-7 scale has been suggested in previous studies such as Beard and Björgvinsson [50], where Items 1, 2, 3, and 7 belonged to the cognitive and emotional component of anxiety and 4, 5, and 6 to the somatic component. This points to the result that machine learning algorithms employed to generate MHSS are more sensitive in picking up the emotional/cognitive component of anxiety.

### Study Implications

The MHSS for anxiety has the potential to serve as a complementary continuous metric to the GAD-7 questionnaire as well as clinical assessment of anxiety disorder. This metric has the advantage of being able to monitor daily anxiety levels with no respondent burden. This enables the use of smartphone-based sensing to overcome any “state-of-mind” biases. Given the metric’s sensitivity to the emotional/cognitive component of anxiety, it can help in overcoming those undiagnosed cases where somatic symptoms of anxiety result in a conflict in diagnosis. This is especially useful in cases where there is an overlap of physical symptoms (shortness of breath or palpitations) and cognitive symptoms (such as insomnia, restlessness) as well as an overlap with depression [9,19]. Another potential use for MHSS is outlining and differentiating the risk of comorbidities. Anxiety disorders are mostly comorbid with depression. A recent study using the same Behavidence research app was able to predict depression severity with the MHSS for depression. Choudhary et al [26] found that machine learning models that generated an MHSS for depression had high accuracy metrics (≥89%) and were able to distinguish between users with depression and those without. Coupled with the findings of this study, MHSS can distinguish between comorbid depression and anxiety, thereby improving clinical decision making.

### Limitations and Future Work of the Study

One of the limitations of the study was that the GAD-7 questionnaire was collected at only 1 time point during the study. In this study the sample size was average, with unequal amounts of gender proportions and education background, which can affect the generalizability of the study, as GAD is a very commonly observed phenomenon. Although the study had almost equal proportions of mild, moderate, and severe groups of anxiety, this was an online recruited sample. With accurate model metrics, further studies should aim for having clinical samples and populations. Therefore, future models should focus on recruiting larger sample sizes and clinical populations to further test the applicability of such findings. Although the machine learning models indicate a higher accuracy of the GAD-7 binary model, the MHSS may have different thresholds for various levels of anxiety severity, which should be subjected to further research. Given the existence of comorbidities, particularly depression, a dedicated study to assess the correlation between MHSS for depression and MHSS for anxiety could generate valuable insights and shed light on how different interventions may be impactful.

### Conclusion

The lack of access to mental health care can be addressed through the ubiquitously available smartphone and the development of passive and widely available screening technologies for detecting the most common mental health disorders. Objective smartphone-collected data contain enough information about an individual’s digital behavior to infer his/her mental states and screen for anxiety, and is a technology that provides remote, longitudinal, and continuous monitoring as an integrative and agile solution. Machine learning serves as an effective technique to mine such big data to derive accurate biomarkers for mental health conditions such as anxiety.

## Appendix

Multimedia Appendix 1 The list of 34 features mined from the data.

Multimedia Appendix 2 Tukey post hoc test for pairwise comparison of the top 3 digital features from the GAD-7 multiclass XGBoost model with Cohen *d* effect size. GAD-7: 7-item Generalized Anxiety Disorder scale.

## Abbreviations

GAD: generalized anxiety disorder
GAD-7: 7-item Generalized Anxiety Disorder Scale
GDPR: General Data Protection Rule
HIPAA: Health Insurance Portability and Accountability Act
MHSS: Mental Health Similarity Score
RMSE: root-mean-squared error
SSL/TLS: secure socket layer/transport layer security
UTC: Coordinated Universal Time
