# Supplementary material for: A Machine Learning Approach for Continuous Mining of Nonidentifiable Smartphone Data to Create a Novel Digital Biomarker Detecting Generalized Anxiety Disorder: Prospective Cohort Study
Source: JMIR Med Inform. 2022 Aug 30;10(8):e38943. doi: 10.2196/38943 (PMC9472035; doi:10.2196/38943)
Supplement: Multimedia Appendix 1 [file medinform_v10i8e38943_app1.pdf]

**Feature**	**Definition**
Mean session	Average session length a user interacts with their mobile device within a 24 hour period (minutes)
Total session	Sum of session lengths a user interacts with their mobile device within a 24 hour period (minutes)
Number of opens	Number of times a user opens an app within a 24 hour period
Sleep	Longest gap time a user is not interacting with their phone within a 24 hour period (minutes)
Average gap	Average length of time a user is not interacting with their phone within a 24 hour period (minutes)
App 0	Total time a user spent on apps that fall into unofficial or unregulated applications within a 24 hour period (minutes)
App 1	Total time a user spent on apps that fall into social interaction applications within a 24 hour period (minutes)
App 2	Total time a user spent on apps that fall into passive information consumption applications within a 24 hour period (minutes)
App 3	Total time a user spent on apps that fall into active messaging and communications applications within a 24 hour period (minutes)
App 5	Total time a user spent on apps that fall into education applications within a 24 hour period (minutes)
App 6	Total time a user spent on apps that fall into general utilities applications within a 24 hour period (minutes)
App 7	Total time a user spent on apps that fall into recreational and

	photo processing applications within a 24 hour period (minutes)
App 8	Total time a user spent on apps that fall into commerce applications within a 24 hour period (minutes)
App 9	Total time a user spent on apps that fall into health and fitness applications within a 24 hour period (minutes)
App 10	Total time a user spent on apps that fall into gaming applications within a 24 hour period (minutes)
App 11	Total time a user spent on apps that fall into miscellaneous and additional passive recreational applications within a 24 hour period (minutes)
App 1- Number of opens	Number of times a user opened apps that fall into social interaction applications within a 24 hour period
App 2- Number of opens	Number of times a user opened apps that fall into passive information consumption applications within a 24 hour period
App 3- Number of opens	Number of times a user opened apps that fall into active messaging and communications applications within a 24 hour period
App 5- Number of opens	Number of times a user opened apps that fall into education applications within a 24 hour period
App 6- Number of opens	Number of times a user opened apps that fall into general utilities applications within a 24 hour period
App 7- Number of opens	Number of times a user opened apps that fall into recreational and photo processing applications within a 24 hour period
App 8- Number of opens	Number of times a user opened apps that fall into commerce applications within a 24 hour period

App 9- Number of opens	Number of times a user opened apps that fall into health and fitness applications within a 24 hour period
App 10- Number of opens	Number of times a user opened apps that fall into gaming applications within a 24 hour period
App 1- Upper Limit	Number of times a user opened apps that fall into social interaction applications and had session times greater than the average session time of that app category within a 24 hour period
App 2- Upper Limit	Number of times a user opened apps that fall into passive information consumption applications and had session times greater than the average session time of that app category within a 24 hour period
App 3- Upper Limit	Number of times a user opened apps that fall into active messaging and communications applications and had session times greater than the average session time of that app category within a 24 hour period
App 5- Upper Limit	Number of times a user opened apps that fall into education applications and had session times greater than the average session time of that app category within a 24 hour period
App 6- Upper Limit	Number of times a user opened apps that fall into general utilities applications and had session times greater than the average session time of that app category within a 24 hour period
App 7- Upper Limit	Number of times a user opened apps that fall into recreational and photo processing applications and had session times greater than the average session time of that app category within a 24 hour period
App 8- Upper Limit	Number of times a user opened apps that fall into commerce

	applications and had session times greater than the average session time of that app category within a 24 hour period
App 9- Upper Limit	Number of times a user opened apps that fall into health and fitness applications and had session times greater than the average session time of that app category within a 24 hour period
App 10- Upper Limit	Number of times a user opened apps that fall into gaming applications and had session times greater than the average session time of that app category within a 24 hour period
