# Supplementary material for: A Machine Learning Approach for Continuous Mining of Nonidentifiable Smartphone Data to Create a Novel Digital Biomarker Detecting Generalized Anxiety Disorder: Prospective Cohort Study
Source: JMIR Med Inform. 2022 Aug 30;10(8):e38943. doi: 10.2196/38943 (PMC9472035; doi:10.2196/38943)
Supplement: Multimedia Appendix 2 [file medinform_v10i8e38943_app2.docx]

| Features | Cohort (mean) | P-value | Cohen’s d |
| --- | --- | --- | --- |
| App Category 2 opens, total number of times  Passive Information Consumption’ applications were opened (count) | | | |
|  | None (0.51) | *P<.001* | -0.03 |
|  | Mild (3.82) | *P<.001* | 0.75 |
|  | Moderate (0.59) | *P<.001* | -0.26 |
|  | Severe (0.43) | *P<.001* | -0.32 |
| App Category 2, average session time on  Passive information consumption applications (minutes) | | | |
|  | None (0.22) | *P=0.46* | -0.08 |
|  | Mild (0.40) | *P<.001* | 0.28 |
|  | Moderate (0.24) | *P=0.73* | -0.01 |
|  | Severe (0.08) | *P<.001* | -0.28 |
| App Category 10 upper, total number of times ‘Games’ were opened with session lengths greater than one standard deviation from the mean (count) | | | |
|  | None (1.61) | *P<.001* | 0.18 |
|  | Mild (0.24) | *P<.001* | -0.52 |
|  | Moderate (2.09) | *P<.001* | 0.55 |
|  | Severe (0.77) | *P<.001* | -0.16 |
